# Supplementary material for: Contact-dependent traits in Pseudomonas syringae B728a
Source: PLoS One. 2021 Feb 11;16(2):e0241655. doi: 10.1371/journal.pone.0241655 (PMC7877591; doi:10.1371/journal.pone.0241655)
Supplement: S1 Table — Number of sequencing reads obtained for the various replicate samples of Pseudomonas syringae B728a cells recovered 2 hours after application to filters or from cells in broth cultures used to inoculate the filters. (PDF) [file pone.0241655.s001.pdf]

Condition	Replicate	Number of sequencing gene reads
Broth	1	46,632,916
Broth	2	41,862,801
Broth	3	32,037,241
Filter	1	47,066,373
Filter	2	47,864,071
Filter	3	20,417,986
