# Supplementary figures and images for: Contact-dependent traits in Pseudomonas syringae B728a
Source: PLoS One. 2021 Feb 11;16(2):e0241655. doi: 10.1371/journal.pone.0241655 (PMC7877591; doi:10.1371/journal.pone.0241655)

**Log<sub>2</sub> Fold Change**

4  
2  
0  
-2  
-4

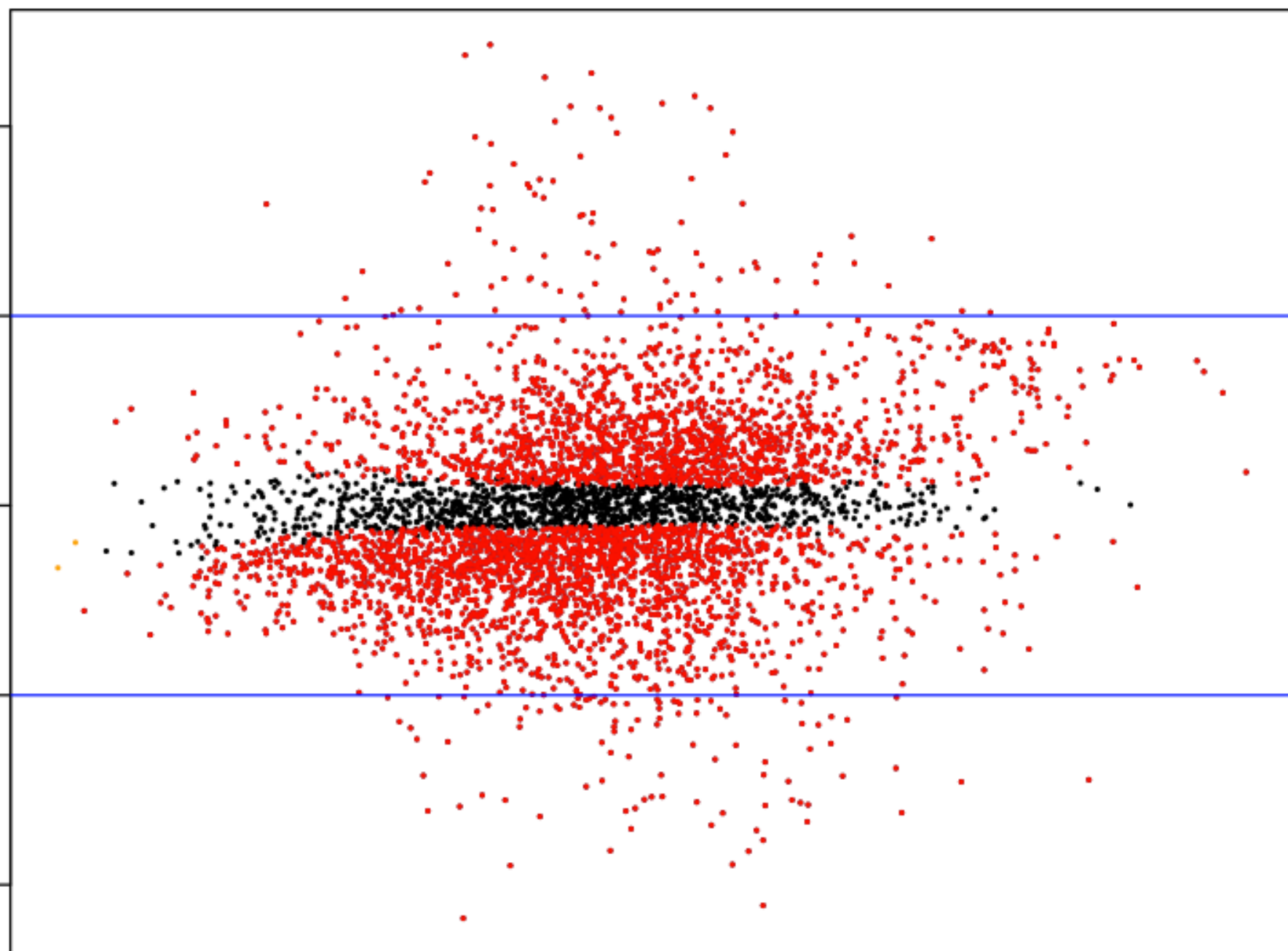

0

5

10

**Average log<sub>2</sub>CPM**

Supplement: S1 Fig — Smear plot documenting the differential expression of genes in Pseudomonas syringae B728a 2 hours after inoculation onto filter surfaces compared to that in planktonic cells as a function of their levels of expression. Shown is the proportion of the genes that were differentially expressed on the filter surface (red) and those not differentially expressed on a surface (black). (PDF) [file pone.0241655.s002.pdf]

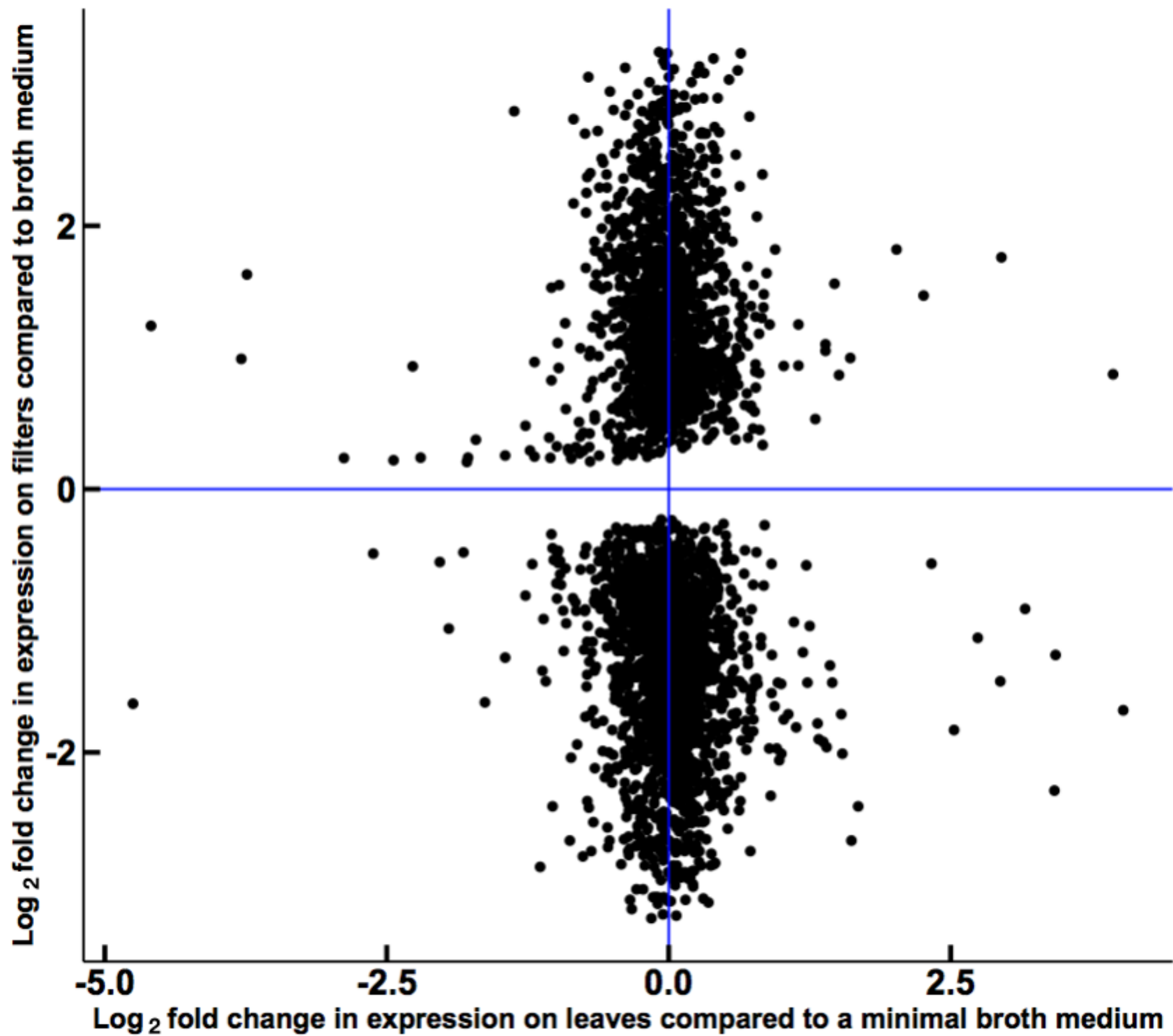

Supplement: S2 Fig — Differential gene expression of genes of P. syringae B728a in cells recovered after growth on bean leaf surfaces for 3 days compared with that in a minimal broth medium in the study of Yu et al. [26] with that of the differential expression of those genes in a rich broth medium compared with that of cells transferred to a membrane for 2 hours in this study. Shown is the log2 fold differential expression in each study. Note that the expression of 5 genes having exceptionally large differential expression in at least one setting has been omitted to enable better illustration of the remaining genes. (PDF) [file pone.0241655.s003.pdf]

Expression

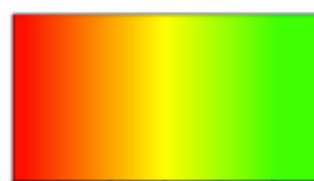

-4 -2 0 2 4

Log<sub>2</sub> Fold

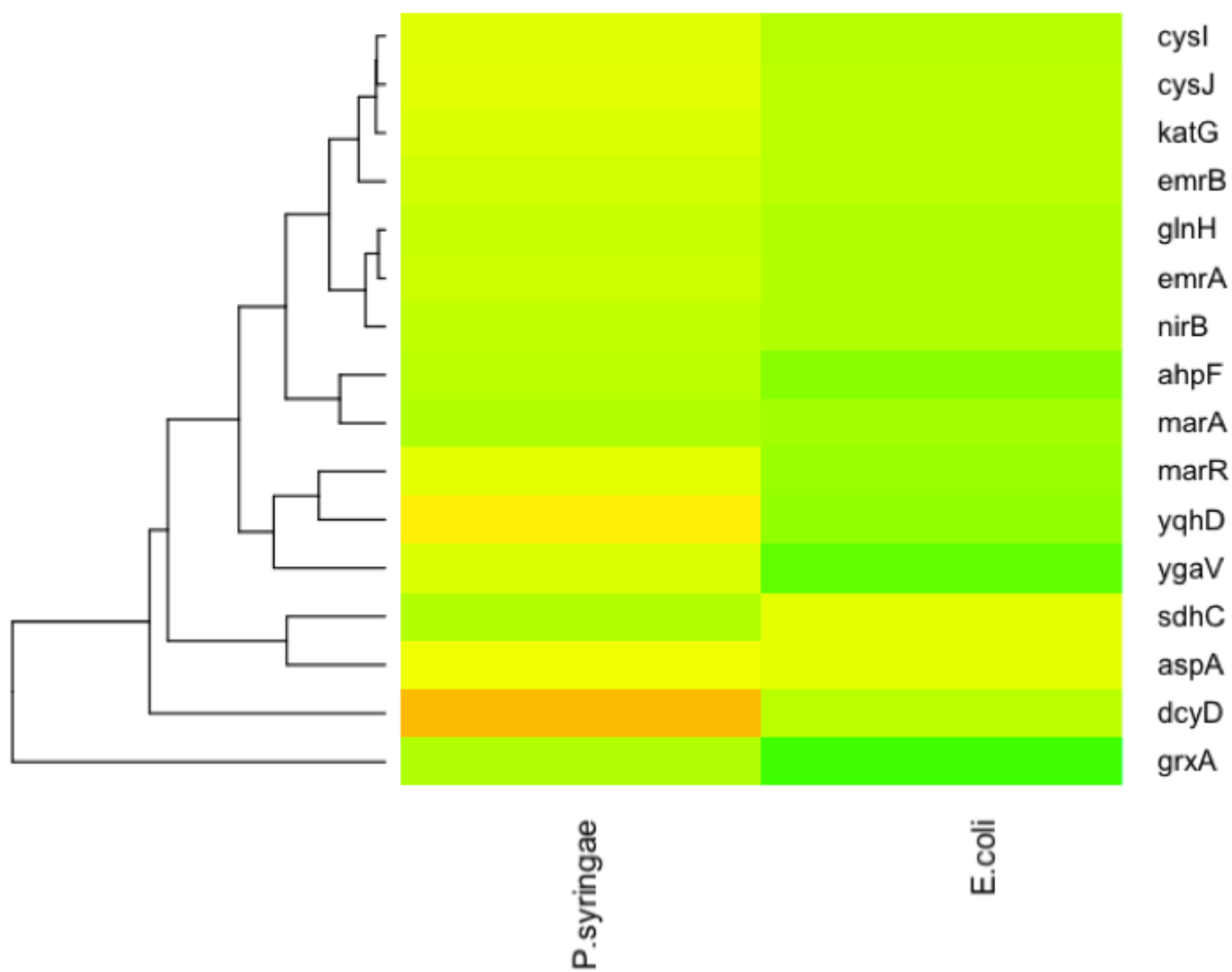

Supplement: S3 Fig — Comparison of differential expression of homologs of genes in Pseudomonas syringae B728a 2 hours after application to membrane surfaces and of E. coli CSH50 1 hour after attachment to mannose agarose beads in the study of Bhomkar et al. [13]. Differential up-regulation of genes is shown in green, down-regulation is depicted in red, and no change in expression is shown in yellow. (PDF) [file pone.0241655.s004.pdf]

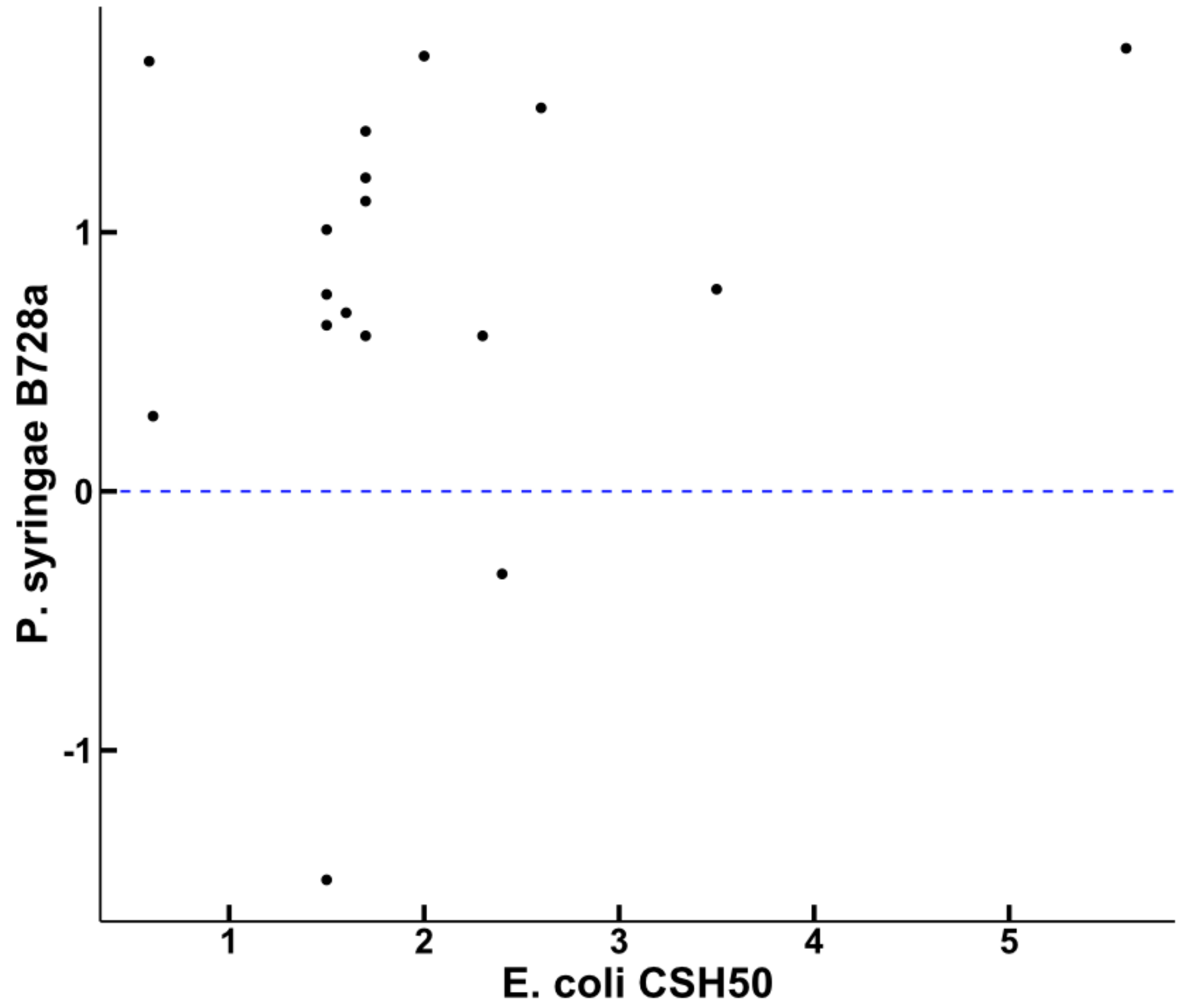

Supplement: S4 Fig — Relationship between magnitude (fold change) of differential expression of gene homologs in Pseudomonas syringae B728a 2 hours after transfer to a filter surface and that of E. coli CSH50 1 hour after attachment on agarose beads in the study of Bhomkar et al. [13]. (PDF) [file pone.0241655.s005.pdf]

*P. syringae* B728a

*P. aeruginosa* UCBPP-PA14

826

62

28

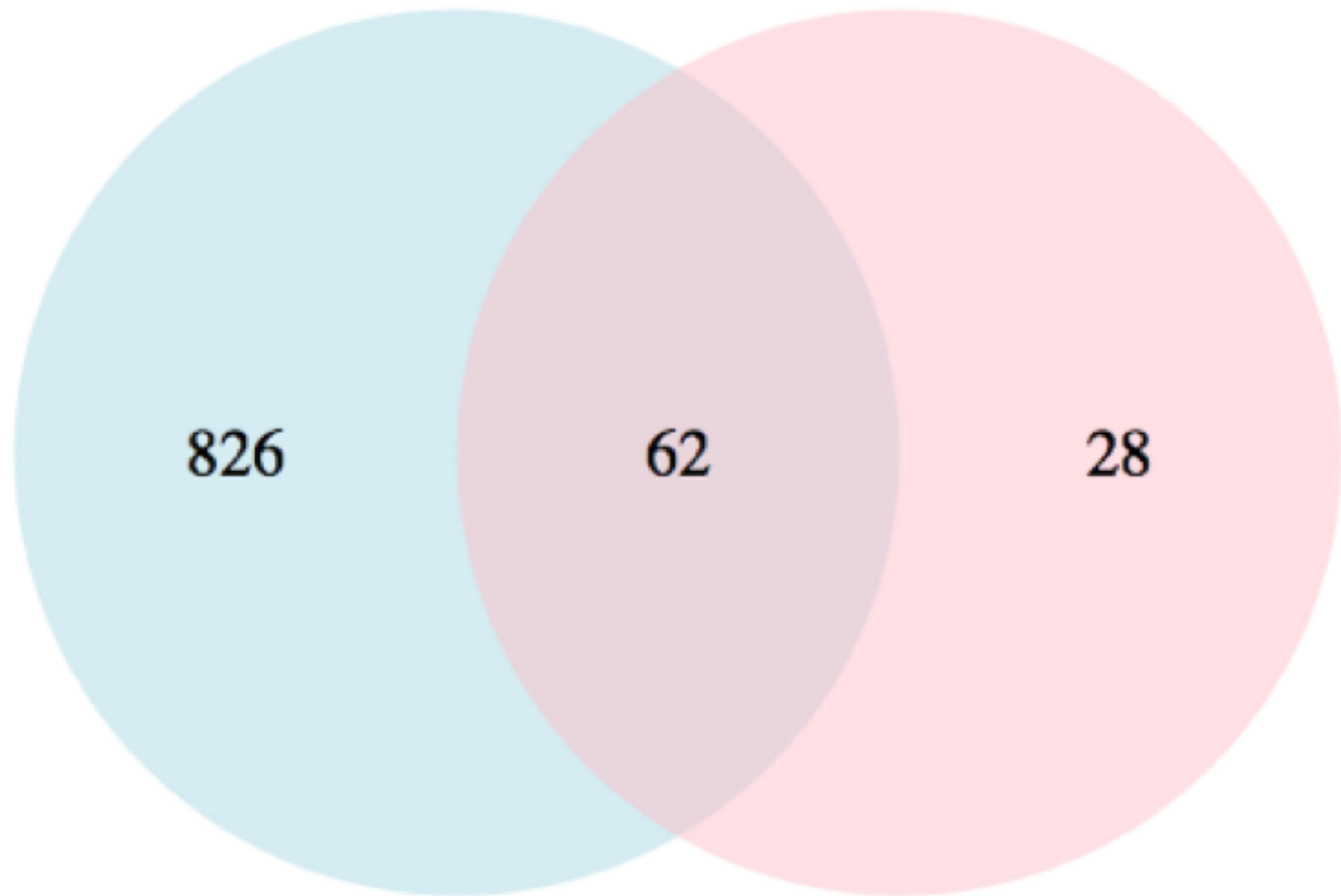

Supplement: S5 Fig — Comparison of the number of gene homologs in Pseudomonas syringae B728a (blue) and Pseudomonas aeruginosa UCBPP-PA14 (red) that were up-regulated when transferred to filter surfaces for 2 hours or were attached to a glass surface for 1 hour in the study of Siryaporn et al. [31], respectively. Using the hypergeometric distribution, this overlap was determined to be significant with a p-value of 1.65E-05 [22]. (PDF) [file pone.0241655.s006.pdf]

Expression

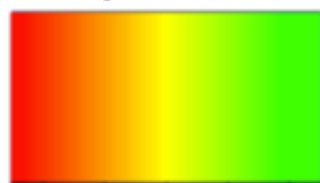

-2 -1 0 1 2

Log<sub>2</sub> Fold

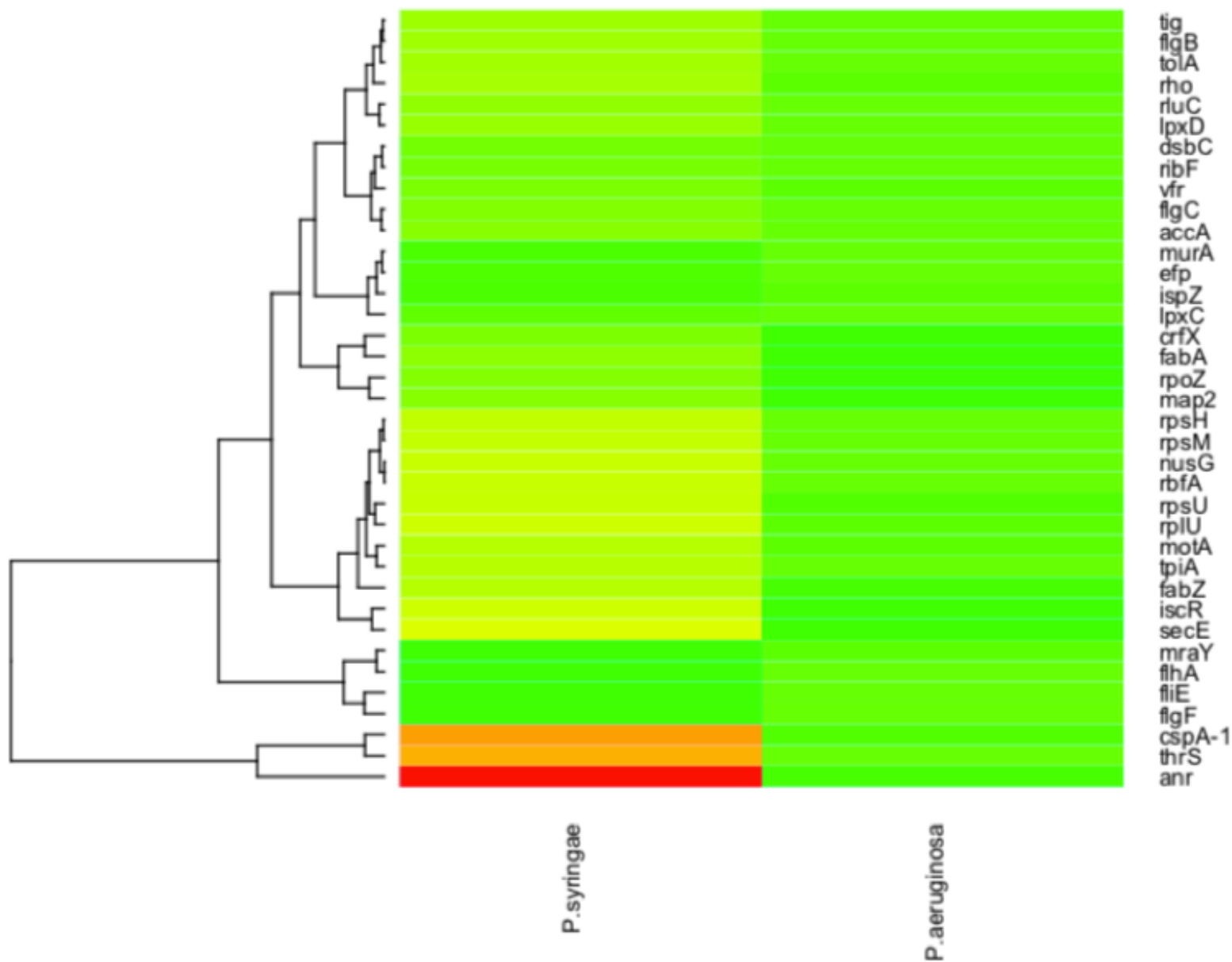

Supplement: S6 Fig — Comparison of differential expression of homologs of genes in Pseudomonas syringae B728a 2 hours after application to membrane surfaces and of Pseudomonas aeruginosa UCBPP-PA14 wild-type cells attached to a glass surface for 1 hour in the study of Siryaporn et al. [31]. Differential up-regulation of genes is shown in green, down-regulation is depicted in red, and no change in expression is shown in yellow. (PDF) [file pone.0241655.s007.pdf]
